# Analysis of Resistance to Wind Suction of Flat Roof Coverings Glued with Polyurethane Adhesives

**DOI:** 10.3390/ma16227135

**Published:** 2023-11-12

**Authors:** Barbara Francke, Jarosław Szulc, Jan Sieczkowski, Artur Piekarczuk, Joanna Witkowska Dobrev, Krzysztof Schabowicz

**Affiliations:** 1 Department of Mechanics and Building Constructions, Institute of Civil Engineering, Warsaw University of Life Sciences-SGGW, Nowoursynowska 159, 02-787 Warsaw, Poland; joanna_witkowska@sggw.edu.pl; 2 Building Research Institute, Filtrowa 1, 00-611 Warsaw, Poland; j.szulc@itb.pl (J.S.); j.sieczkowski@itb.pl (J.S.); a.piekarczuk@itb.pl (A.P.); 3 Faculty of Civil Engineering, Wrocław University of Science and Technology, Wybrzeże Wyspiańskiego 27, 50-370 Wrocław, Poland; krzysztof.schabowicz@pwr.edu.pl

**Keywords:** flat roof coverings, flexible sheets for waterproofing, polyurethane adhesives, resistance to wind suction, fatigue laboratory tests, numerical simulations

## Abstract

The article analyses the impact of wind suction on roof coverings glued with polyurethane adhesives to flat roofs, i.e., roofs with an up to 20% slope. The impact of the cyclical wind was simulated in fatigue tests, gradually increasing the test pressure in repeated sequences until the first delamination occurred. The tests were carried out for eight test sets, with concrete and trapezoidal sheets used as a construction substrate, on whose surface thermal insulation layers were glued with polyurethane adhesive; the thermal insulation layers were EPS (expanded polystyrene) and PIR (polymer mainly of polyisocyanurate groups), respectively, followed by flexible sheets, i.e., a laminated PVC membrane (polyvinylchloride) and an EPDM (terpolymer of ethylene, propylene and a diene with a residual unsaturated portion of diene in the side chain)-type rubber-based membrane. The test results were compared with the functional requirements determined with computational simulation methods for the maximum wind load values on the example of wind loads for Poland. The tests confirmed that some polyurethane adhesives could ensure the operation of flexible sheets used as flat roof coverings that are failure-free from the point of view of resistance to wind suction.

## 1. Introduction

Roof coverings with traditionally arranged layers, and on roofs with up to 20% (approx. 11.5°) slopes, are broadly used in housing and industrial construction. Roof coverings on such roofs are usually made of flexible sheets for waterproofing, i.e., bitumen sheets with reinforcement or plastic and rubber membranes, laid over a thermal insulation attached to a substrate made of concrete or trapezoidal sheeting. In both cases, such layers can be mechanically fixed or glued to the layer below and to the construction layer. Two fixing methods are used: mechanical fasteners (anchors) and glue with adequate adhesives or glues [1]. Covering products can be glued by welding with liquid gas (propane and butane) burners (e.g., for heat sealing roofing reinforced bitumen sheets) or hot air (e.g., plastic and rubber membranes). The interlayer adhesion is affected by the following four factors [2,3]:

- The type and power of the chemical interaction with the glued surfaces—adhesion—if the adhesive reacts chemically with the substrate forming chemical bonds with it; typically, such a weld is more resistant to being formed by adhesives that penetrate only into the glued surface;
- The penetration depth into the glued material—the higher, the better, but if the adhesive penetrates too deep, it can damage the glued material’s structure; the penetration depth can be increased by raising the roughness of the glued material’s surface;
- The mechanical strength of the adhesive layer, cohesion, depends on the chemical structure of the adhesive;
- The size and shape—the larger the adhesive bond area and the more irregular its shape, the stronger it becomes.

Work has been carried out on extending the group of adhesive materials suitable for use in roof coverings. It includes attempts to use polyurethane adhesives to glue roof covering made of plastic membranes and roofing reinforced bitumen sheets, as well as thermal insulation layers where mechanical fixing or open-flame glueing is replaced. Studies have shown that polyurethane (PU) adhesives form a strong bond with several substrates and perform better under quasistatic, dynamic, impact and cyclic loads and other environmental conditions than other commercial adhesives, such as epoxies [4]. Good adhesion and other outstanding properties of PU adhesives enable their extensive use in several applications, including all types of sandwich composites, constructions, tools, vehicle repairs, thin plate bonding, weld and rivet bonding, bonding of reinforced plastic and sheet moulding compounds [5,6]. As an adhesive, polyurethane (PU) can effectively wet the surfaces of many substrates. Its low viscosity allows it to spread through porous substrates. Moreover, polyurethane forms hydrogen bonds with several substrates and covalent bonds with substrates that contain active hydrogen. It also exhibits good toughness and resistance to water and a broad range of chemicals. Manufacturers of polyurethane (PU) have been offering solutions to glue thermal insulation layers within the façade, but in this case, in Europe, additional mechanical fixing (anchors) is still required [7]. Polyurethane glues are adhesives with a chemical hardening mechanism because they react with water in the material or environment, for example, the air. This means that two effects co-occur, i.e., liquid adhesive hardening and a chemical reaction that additionally affects the glueing properties. They are reactive adhesives, which means that when the adhesive is exposed to high temperatures after crosslinking, its state of aggregation does not change, i.e., the adhesive does not melt, which is a prerequisite for a product used to glue roofing covering at working temperatures up to +70 °C. In terms of the chemical basis of polyurethane glues, called polyurethane (PU), they are polymers formed due to the addition polymerisation of multifunctional isocyanates with polyols. Polyurethane (PU) adhesives contain several urethane groups in their molecular structural backbone. They are obtained by step-growth polymerisation (polyaddition) from three main components: isocyanates, polyols or prepolymers (active hydrogen compounds) and low-molecular-weight chain extenders or crosslinkers (diamines or diols) [8]. In addition, catalysts (such as tertiary amines) and additives (e.g., drying agents, antioxidants, UV inhibitors, fillers or colourants) can also be added, thus contributing to significant differences between several types of PU products. Double-component polyurethane adhesives are based on the reaction of multisubstantial alcohols (polyols) with multifunctional isocyanates. Monocomponent polyurethane adhesives are made of double-component adhesives so that polyol is kept to react with the abundance of isocyanate. These are sensitive to water, alcohols, amines, acids, etc. Adhesives are set under the influence of air humidity in three steps. In the first step, isocyanate reacts with water, and unstable carbamic acid is formed. The second step involves the disintegration of carbamic acid into carbonic oxide and amine. Finally, the third step is a reaction of amine and isocyanate and polyurethane linkage creation [8,9,10]. In most cases, monocomponent polyurethane adhesives are used to adhere to flat roofs.

Polyurethane adhesive-bonded roofing is exposed to specific service loads. Roof coverings should be able to bear the loads resulting from UV radiation, in the presence of positive and negative temperatures, water and moisture without damage [1]. They should also safely carry all mechanical loads, including wind suction, particularly relevant for flat roofs.

On nearly all continents, unprecedented climate impacts have become more common, resulting from the greenhouse effect and excessive carbon dioxide emissions to the atmosphere. Researchers investigate the complex relationship between spatial composition and building typology, on the one hand, and thermal and climatic conditions within and between buildings, on the other hand [10]. Climate change consequently leads to the risk of the frequent occurrence of strong winds and violent thunderstorms, negatively affecting the safety of building structures and their surrounding areas. When the wind blows, buildings pose an obstacle around which it needs to flow. The flow around the object is a frequently solved problem [11,12]. Furthermore, scientists often discuss the flow around cylindrical objects or structures such as cooling towers, chimneys [13], buildings of circular shapes, cable car pylons, bridge structures [14,15,16], offshore structures, air-cooled heat exchangers [16], storage tanks and other industrial buildings [17] and their structural components. More detailed knowledge of the flow field and the effects of wind flow on objects is applied in civil engineering [18] and wind engineering [19,20,21,22]. Wind flow problems can be related to the buildings’ layouts and shapes of buildings [23,24], as well as the type of the cladding [13], the shape of balconies [11], etc. A large vortex with relatively low airflow velocity is formed behind the building; vortexes are also formed behind the windward edges [25,26,27,28]. The destructive impact of the wind does not always result from its high velocity. Construction damage might occur due to various aerodynamic phenomena observed at a relatively low wind flow velocity.

In order to reduce as much as possible the risk of de-bonded roof covering due to wind impact, effective fixing of all roof layers to the substrate is necessary. This applies to roof coverings and thermal insulation layers. This suggests that ensuring chemical compatibility between the glued material and the adhesive contributes significantly to achieving adhesion, guaranteeing the resistance of the roof cover to wind suction.

The aim of tests discussed in this paper was to confirm that polyurethane adhesives used for the adhesive of PVC (polyvinylchloride) and EPDM (terpolymer of ethylene, propylene and a diene with a residual unsaturated portion of diene in the side chain) membranes on flat roofs with an up to 20% slope ensure high resistance of complete systems to wind suction. The technical literature mentions scientific reports on attempts to replace mechanical fixing of roof covering layers with polyurethane adhesives, but the resistance of such systems to wind suction in tests simulating cyclic impacts has not been determined so far. Previous studies used indirect methods to assess the referenced property, including but not limited to resistance perpendicular to the surface [7]. However, this method does not consider the accumulation of fatigue caused by wind impact, which means that only one component is defined that completes the destruction process. The European standard EN 17686 [29] solves part of the problem, as it describes the cyclical testing of flexible roof coverings for wind suction and expresses the resistance to wind load of a flexible roof waterproofing system as the characteristic load per square metre. According to the standard, safety and correction factors can be defined by national regulations and/or within European or national application documents. Due to the complex nature of the tests described in the standard, the results of polyurethane adhesives have not been published previously. Facing the needs of the construction community, this paper discusses the results of our own laboratory tests that simulate the behaviour of flexible sheets glued with polyurethane adhesives; the tests simulated cyclic wind suction using a test method modified against that described in EN 17686 [29]. The test results were compared with functional requirements determined with computational simulation methods and identified for the maximum wind load values in Poland. The results indicate that some polyurethane adhesives can ensure the failure-free operation of flexible sheets in flat roof coverings from the viewpoint of their resistance to wind suction. However, it was deemed reasonable for complicated geometry and complex wind load; in order to correctly design covering solutions for a specific structure, it is recommended to compare the results obtained in the laboratory tests with the values obtained in the calculations performed to EN 1991-1-4 [30], supplemented with those determined in CFD numerical simulations. 

## 2. Materials and Methods

### 2.1. Materials

Based on initial elimination tests performed for different roofing systems, conducted by using the pull-off adhesion method on small-sized samples (250 × 250 mm), we chose two polyurethane adhesives for further studies on large samples, which seemed to be common for the presented material groups:

- Product 1—polymerisation of monocomponent polyurethane adhesive under moisture, open time ca. 30 min, complete curing time ca. 90 min, density 1.11 g/cm^3^, dry matter content 99%, thermal resistance from −30 °C to +120 °C, application temperature between +5 °C and +40 °C;
- Product 2—polymerising of monocomponent polyurethane adhesive under moisture (polyurethane prepolymer), skin—over time ca. 80 min, open time ca. 40 min, complete curing time ca. 60 min, thermal resistance between −40 °C and +90 °C, application temperature from 0 °C to +35 °C.

As part of the research, the following auxiliary products were used to make test kits:

➢ Substrates, consisting of two elements for each test specimen, size 1.25 m × 2.5 m, with a gap over the entire length of the test specimen, approximately 4 mm ± 1 mm wide and over the full height of the substrate, placed in parallel to the side of a pressure chamber:
  (a) Concrete slabs, C 30/37;
  (b) Galvanised trapezoidal steel sheets T55.
➢ Thermal insulation materials:
  (a) PIR ETX 50 boards (rigid cellular polymeric product mainly of polyisocyanurate groups), 50 mm thick, consisting of a thermal insulation core made of rigid PIR foam, density 30 kg/m^3^, covered on one side with glass fibre tissue (ETX); hardness and resistance to damage σ10 = 150 kPa, λ = 0.027 W/mK;
  (b) EPS 100 boards (expanded polystyrene), 50 mm thick, compressive stress: 100 kPa, flexural strength: 150 kPa, λ = 0.036 W/mK.
➢ Roof covering materials:
  (a) PVC membrane (polyvinylchloride membrane)—roofing membrane made of pliable PVC, 1.2 mm wide, laminated on the underside of the nonwoven polyester roll, tensile mechanical characteristics 1100 N/50 mm, at elongation ≥ 15%, flexibility at low temperature −30 °C.
  (b) Laminated EPDM membrane (terpolymer of ethylene, propylene and a diene with residual unsaturated portion of diene in the side chain membrane), 2.3 mm thick, laminated with non-reinforced synthetic membrane formed from two EPDM layers with a total thickness of 1.3 mm. The membrane is laminated with nonwoven polyester fabric, 160 g/m^2^ mass per unit area. Maximum tensile force of membrane 400 N/50 mm, at elongation < 40%, flexibility at low temperature < −40 °C.
  (c) EPDM membrane—not laminated, with non-reinforced synthetic membrane formed from two EPDM layers (i.e., synthetic rubber—ethylene-propylene-diene-monomer) with a total thickness of 2.0 mm.
  (d) Bituminous primer, bitumen content ca. 65%, density: 0.85–0.95 kg/L, VOC content 350 g/L.
  (e) Vapour barrier—elastomeric reinforced bitumen sheet, 3.8 mm thick, with a glass fabric insert with aluminium coating. Maximum tensile force 500/400 N/50 mm (+/−100/100), at elongation 4/4% (+/−2/2), flexibility at low temperature −15 °C.

After applying polyurethane adhesive, air humidity was increased—by producing a water mist around the samples—in the room where the test samples were kept. The layer was glued around the base 5–10 min after applying a coat of adhesive to the substrate.

Each test set consisted of the following layers (counting in stacking order):

(a) For concrete substrates:
  - Concrete slabs, C 30/37 primed with a bitumen liquid + vapour control layer (reinforced bitumen sheet) glued to the substrate over the surface;
  - Thermal insulation boards listed in Table 1 in the third column;
  - Covering layers listed in Table 1 in the fourth column.
(b) For substrates of trapezoidal sheets:
  - Galvanised trapezoidal steel sheet T55;
  - Thermal insulation boards listed in Table 1 in the third column;
  - Covering layers listed in Table 1 in the fourth column.

The test sets are described in detail in Table 1. 

**Table 1:** Summary of the test sets.

Set No.	The Arrangement of the Layers (Stacking Order)		
	Substrate	Thermal Insulation Boards	Covering Layer
Set I	Concrete primed with a bitumen liquid + vapour control layer (reinforced bitumen sheet) glued to the substrate over the surface.	PIR ETX, 50 mm thick, glued to the substrate with polyurethane adhesive—product 2, strips applied every 15–20 cm.	Laminated PVC membrane, glued to the substrate with polyurethane adhesive—product 1, all over the surface
Set III			Laminated EPDM membrane, 2.3 mm thick, glued to the substrate with polyurethane adhesive—product 1, all over the surface
Set II		EPS 100, 50 mm thick, glued to the substrate with polyurethane adhesive product 2, strips applied every 15–20 cm.	Laminated PVC membrane, glued to the substrate with polyurethane adhesive—product 1, all over the surface
Set IV			Laminated EPDM membrane, 2.3 mm thick, glued to the substrate with polyurethane adhesive—product 1, all over the surface
Set V	Galvanised trapezoidal steel sheet T55.	PIR ETX 50, 50 mm thick, glued to the substrate with polyurethane glue—product 2, two strips on each upper flange of the sheet steel (along the edge).	Laminated PVC membrane, glued to the substrate with polyurethane adhesive—product 1, all over the surface
Set VII			EPDM membrane (not laminated), 2.0 mm thick, glued to the substrate with polyurethane adhesive—product 1, all over the surface
Set VI		EPS 100, 50 mm thick, glued to the substrate with polyurethane adhesive—product 2, two strips on each upper flange of the sheet steel (along the edge).	Laminated PVC membrane, glued to the substrate with polyurethane adhesive—product 1, all over the surface
Set VIII			EPDM membrane (not laminated), 2.0 mm thick, glued to the substrate with polyurethane adhesive—product 1, all over the surface

### 2.2. Methods

#### 2.2.1. Laboratory Tests

**Figure 1:**
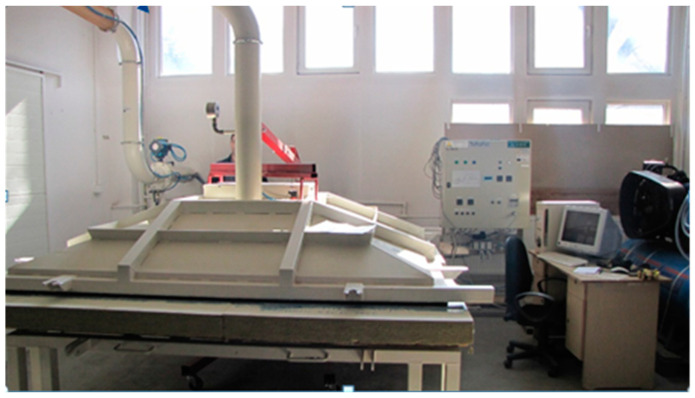
Wind suction resistance testing chamber.

The resistance to wind suction was tested in a chamber shown in Figure 1. 

**Figure 2:**
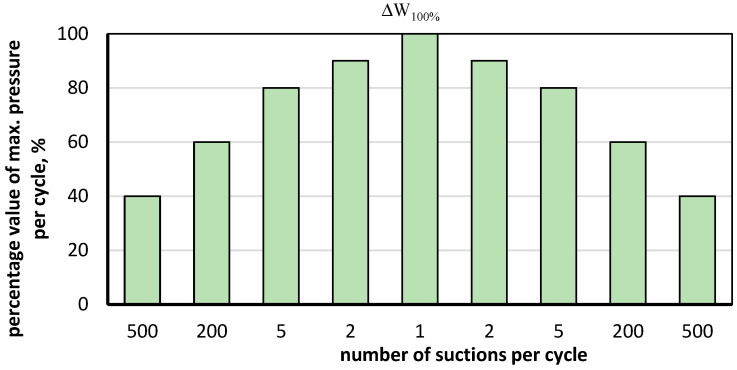
Pressure distribution in the chamber during one test cycle, where ∆W_100%_—maximum peak load per cycle.

The test involved exposing the test set described in Table 1 to cyclic wind suction in periods summarised in Table 2. A single test cycle included gradually reaching successive pressure values mentioned in Table 2. The method of raising the pressure during the test cycle is shown in Figure 2. 

Figure 2 shows how the pressure in the chamber is successively increased to the set value and then decreased in each test cycle using the principle of multiple repetitions of successive pressure values representing a percentage of the maximum value (according to the values shown in Figure 2 for successive cycles). The maximum pressure value in a complete test cycle is taken in turn as the value listed in the second column in Table 2. A complete test cycle in the range from 1 kPa to 4 kPa consists of a 4-fold repetition of the cycle shown in Figure 2, with a maximum value ∆W_100%_ 1 kPa, followed by one complete cycle (as above) with maximum values of successively 1.5 kPa, 2.0 kPa, 2.5 kPa, 3.0 kPa, 3.5 kPa and 4.0 kPa. If the set was not damaged up to the value of 4.0 kPa, the pressure in the test chamber was further increased, according to the rule of reaching and reducing the value in ten cycles, without multiple repetitions, shown in Figure 1 for values up to 4.0 kPa. The test result is the maximum pressure value at which one of the tested roof covering layers is delaminated. Additionally, the nature of the damage was evaluated. 

#### 2.2.2. Numerical Investigation

An analysis [30] of wind load values occurring in European countries (e.g., France, Germany, Poland) confirmed that in the climate zone marked Dfb in Köppen classification, i.e., humid continental mild summer, wet all year, average wind speeds in most areas of Europe are between 24.0 m/s and 27.5 m/s, reaching up to 30 m/s in extreme zones. Of course, wind speed is not the only determinant of the negative impact of wind on a building structure. In addition to the location of a building in a specific wind zone, the shape, height and, in the case of roofs, the distance from the edge zone must also be taken into account. In order to determine the suitability of the polyurethane adhesives for bonding roof coverings to flat roofs using a calculation method, the test results given in point 3.1 were compared with the calculation values for the reference case of wind effects on roof covering, generating the most adverse effects of impact. Reference was made to Köppen’s classification, which based the climate division on mean monthly temperatures and the amount and distribution of annual precipitation, with reference to latitude. For the calculations, wind speeds according to PN EN 1991-1-4 and the National Annex with a value roughly corresponding to the climate zone Dfb were used. The following assumptions were made for the calculations according to PN EN 1991-1-4 and considering the National Polish Appendix (NA) [30,31]:

- A roof surface for a standard industrial structure, at a roof slope α < 11.5° (i.e., 20%).
- Dimensions of the building:
  - Length d = 30.0 m;
  - Width b = 15.0 m;
  - Height h = 10.0 m (typical height of double-storey industrial facilities), an altitude A = 1100 m above ground level.
- Dimension e = min (b, 2 h) = 15.0 m.
- The basic value of the wind base velocity according to EN 1991-1-4 [30]:

v_b,0_ = 22 · [1 + 0.0006·(A − 300)] = 32.56 m/s (1)

- Directional factor (unknown wind direction): c_dir_ = 1.0.
- Seasonal factor: c_season_ = 1.00.
- Wind base velocity: v_b_ = c_dir_·c_season_·v_b,0_ = 32.56 m/s.
- Reference height: z_e_ = h = 10.00 m.
- Terrain category I roughness coefficient:

c_r_(z_e_) = 1.2·(10.0/10)^0.13^ = 1.20

- Coefficient of topographic profile (orography): c_o_(z_e_) = 1.00.

It was verified that these parameters do not differ from the annexes of other European countries.

The orography coefficient includes the impact of slopes or single elevations (but not in hilly or mountainous areas) on wind velocity. This way, it considers the impact of local topographic conditions not covered by typical terrain roughness categories. The impact of the topographic profile was neglected, assuming that the mean windward land slope is lower than 11.5° (approx. 20%). In highly unfavourable scenarios, a building structure can be situated on a single hill, a hill range, a cliff or a scarp slope, which can contribute to increasing the orography coefficient c_o_(z_e_) > 1.00 and a proportional increase in the wind impact force.

- Mean wind velocity:

v_m_(z_e_) = c_r_(z_e_)·c_o_(z_e_)·v_b_ = 39.07 m/s (2)

- Turbulence intensity: I_v_(z_e_) = 0.145.
- Air density:

ϱ = 1.25 · [(20,000 − A)/(20,000 + A)] = 1.12 kg/m^3^ (3)

- Velocity pressure peak value:

q_p_(z_e_) = [1 + 7·I_v_(z_e_)]·(1/2)·ϱ·v_m_^2^(z_e_) = 1720.7 Pa = 1.721 kPa (4)

- Construction coefficient (building less than 15 m high): c_s_c_d_ = 1.000.
- External pressure coefficient c_pe_ = c_pe,10_ = −1.8.

## 3. Results

### 3.1. Results of Laboratory Tests of Wind Suction Resistance

**Figure 3:**
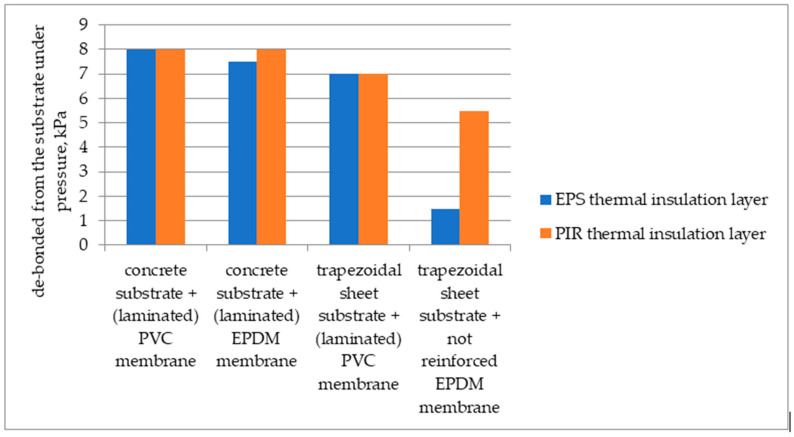
Comparative summary of resistance to wind suction for different test sets—large samples.

The results of the wind suction resistance for the large-sample tests, where roofing materials were glued with polyurethane adhesive, are summarised in Table 3 and Figure 3. 

### 3.2. Results of Numerical Calculations

**Figure 4:**
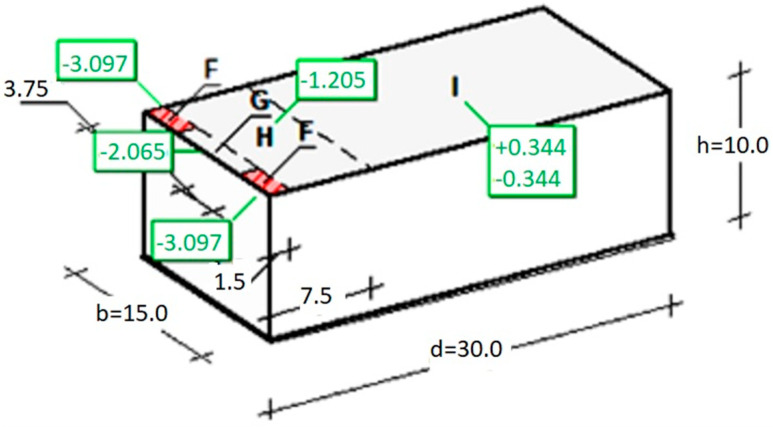
Basic dimensions of a flat roof building and wind load results (suction in kN/m^2^).

For the computational assumptions described in Section 2.2.2, the extreme wind impact force (“−“ means suction) on the external surface (in the corner zone F—as shown in Figure 4) amounts to

F_w,e_ = c_s ∙_ c_d_·q_p_(z_e_)·c_pe_ = 1.000·1.721·(−1.8) = −3.097 kN/m^2^ (5)

## 4. Discussion

Testing the resistance to wind suction of covering sets glued with polyurethane adhesives showed that the adhesive bond between an EPS board and a plastic or rubber membrane roof covering is the weakest spot. Although the covering was glued to the surface of a substrate made of a thermal insulation material substrate, the de-bonding as a result of wind suction simulation occurred at the border between the thermal insulation layer and the roof covering. At the quoted pressure values, no damage was discovered to the adhesive bond formed between the concrete or trapezoidal sheet substrate layer and the thermal insulation layer made of EPS or PIR boards. A certain regularity was observed that the resistance to wind suction for the roof cover laid on thermal insulation material glued to a concrete substrate is higher than for a trapezoidal sheet substrate, as shown in Figure 3, even though the covering layer is separated from the substrate with a thermal insulation board, additionally glued at the contact points between the boards with polyurethane adhesive, that is, product 2.

The distances between the adhesive strips applied to the concrete and trapezoidal sheet substrates are similar, ranging from 15 to 20 cm for the concrete substrate and 20.5 cm for the trapezoidal sheet substrate. The distance between the adhesive strips can be smaller on a trapezoidal sheet because when the adhesive swells, the sheet metal folds are partially filled, considerably reducing the distance between the adhesive strips.

**Figure 5:**
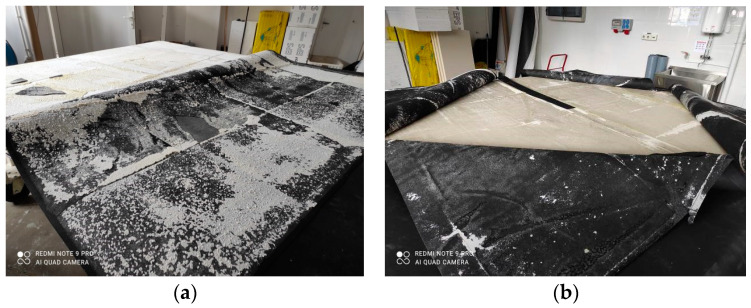
Nature of the roof covering pulling off. (**a**) EPDM laminated to EPS substrate, (**b**) EPDM with no lamination from the EPS substrate.

The variants tested confirm a statement presented in the literature analysis in relation to the increase in the adhesion within the adhesive bond when the adhesive material can partially penetrate the glued layers’ structure. It is evident in the comparison of the wind suction resistance test results for laminated membranes and membranes with no additional bottom layer reinforcement with a nonwoven material. Polyurethane adhesive penetrates the nonwoven material and together they form a rigid and robust layer within which the interlayer cohesion forces do not exceed the adhesion value between the laminating layer and the membrane without compromising adhesion in this plane. De-bonding (pulling off) as a result of suction force impact occurs in the thermal insulation layer (for EPS) or in the thermal insulation layer (for PIR). In this case, the nature of the break seems evident because the PIR board is additionally reinforced superficially with a glass fibre tissue, which contributes to an increase in its resistance to delamination. The differences in the nature of the break mentioned above are shown in Figure 5. 

The test results indicate that some commercial polyurethane adhesives can transfer extreme wind load values after being used in a weld formed at the border between the thermal insulation material and the roof covering on roofs with up to 20% slopes. The computationally determined extreme value of the wind impact force in Poland, F_w,e_ = 3.097 kN/m^2^, is considerably lower than the 4.0 kPa achieved during the simulation of wind suction impact on PVC or EPDM membrane roof covering glued to the EPS or PIR substrate with a random polyurethane adhesive. This means the loads at which the pull-off strength from the substrate was verified (variants I–VII according to Table 3). 

The wind suction value determined in Section 3.2 is a generalised load resulting directly from the computational algorithms and assumptions of standard EN 1991-1-4 [30]. The loads summarised in the standard are commonly used in engineering calculations. It is justified because they ensure reasonably safe, consideration of the most adverse effects of impact. In addition to standards, wind engineering follows other approaches to the phenomenon of the wind load of building structures, e.g., experimental tests in aerodynamic tunnels [32,33,34]. Such tests are the most accurate method to assess the impact of wind impact on structures, but they are still very complicated and require specialised equipment. Computational fluid dynamics (CFD) numerical calculations offer an alternative to experimental tests [35]. They are presented in many scientific papers, and their suitability for use in engineering is discussed in papers [36,37,38]. 

This paper uses a simplified CFD simulation performed with RWIND software version 1.26 (https://www.dlubal.com/en/products) for comparative analysis.

The RWIND software is an engineering tool for simplified CFD analyses and is part of the numerical system. In the analysed cases, simple models of building geometry were adopted, which are located in the fluid domain, that is, directly surrounded by a mesh of volumetric finite elements. The dimensions of the domain are 300 × 150 × 100 m, which, compared to the dimensions of the building, 30 × 15 × 10 m, is an aspect ratio of 1/10. With a well-selected mesh, the correct results can be obtained in a domain with a proportion of 1/5. A large domain significantly lengthens the calculations but allows one to obtain results with an error not exceeding 1%. Hexahedral volume elements are used for meshing. In total, approximately 2 million hexahedral cells with varying degrees of density were used in the task, especially in the influence area. The basic turbulent RANS model (Reynolds averaged Navier–Stokes equations) and the standard K-ε method were adopted in the calculations, with a residual pre-convergence criterion of 0.1%. The analysis was carried out iteratively in 500 steps.

**Figure 6:**
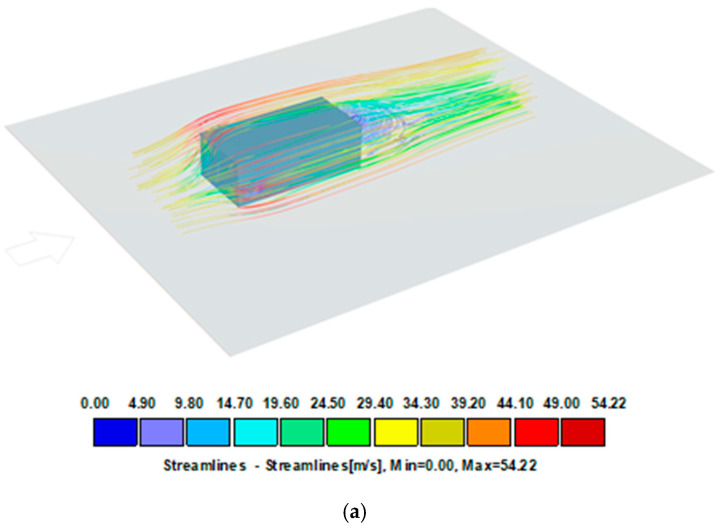

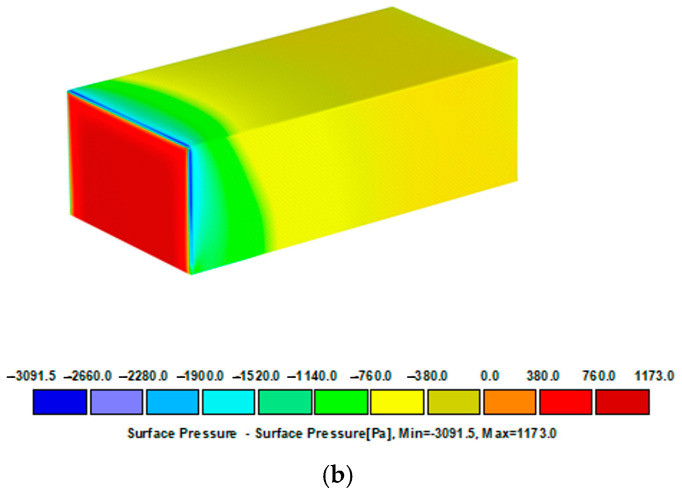
Wind velocity current lines and surface pressure. Charge perpendicular to the front of the building. Project CFD- Model 30 × 15 × 10 m, free stream velocity: 39.07 m/s. (**a**) Streamlines, (**b**) surface pressure.

**Figure 7:**
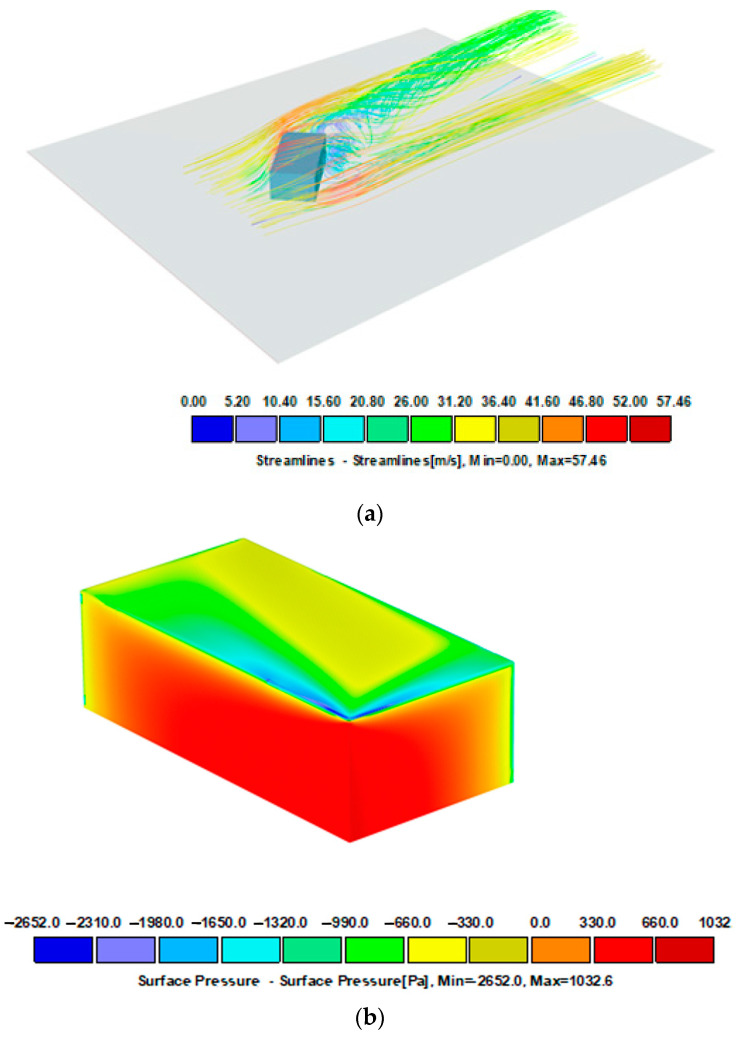
Wind velocity current lines and surface pressure. Modified model. The load rotated by 45°. Project CFD +45—Model 30 × 15 × 10 m, free stream velocity: 39.07 m/s. (**a**) Streamlines, (**b**) surface pressure.

**Figure 8:**
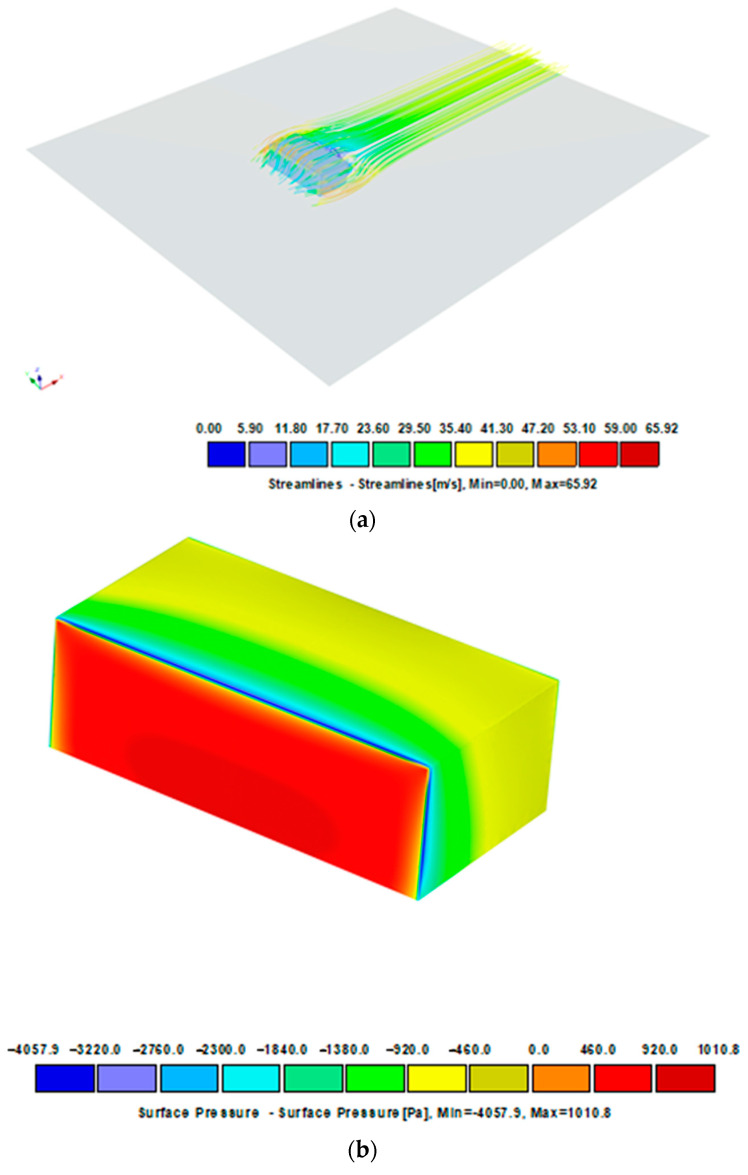
Wind velocity current lines and surface pressure. Charge perpendicular to the side surface. Project CFD +90—Model 30 × 15 × 10 m, free stream velocity: 39.07 m/s. (**a**) Streamlines, (**b**) surface pressure.

With complex building shells, locally increased wind suction values may occur. The simulations shown in Figure 6, Figure 7 and Figure 8 illustrate the pressure differences that can occur in complex building envelopes, allowing them to be compared with the values obtained using the laboratory method described in this paper. A mean wind velocity was taken for the simulation, analogously to the example in item Section 2.2.2. The simplification involves assuming a stationary air flow in the domain (the volume surrounding the tested object). The simulation results for three different wind impact directions are shown in Figure 6, Figure 7 and Figure 8.

Figure 6 shows a model of an object exposed to wind load perpendicularly to the front of the building, the same as in the standard analysis (Figure 4). The pressure distribution on the surfaces has smaller coverage and different zones. However, their values are similar to those quoted in standard calculations. In the referenced system, the pressure value on nearly the entire roof covering surface is ca. 380 Pa (yellow), while its extreme value on the surface amounts to 3091 Pa. When the wind direction is rotated 45 degrees and orientated towards the building’s corner, the pressure distribution is different (Figure 7). The windward walls are exposed to wind pressure. The roof is exposed to wind suction, so the extreme value of 2651 Pa occurs at the edges, which is slightly less than shown in Figure 6. The case is different for the load perpendicular to the building’s side surface of the building (Figure 8). In such an arrangement, the wind suction loads are the highest at the edge and reach ca. 4000 Pa. It should be noted that an extreme load concerns a reasonably small surface adjacent to the edge, which can be attributed to the impact of the local coefficient of external pressure C_pe,1_ = −2.5 (applies to 1 m^2^ area) compared to a larger area, that is, C_pe,10_ = − 1.7 (applies to 10 m^2^ area). 

**Figure 9:**
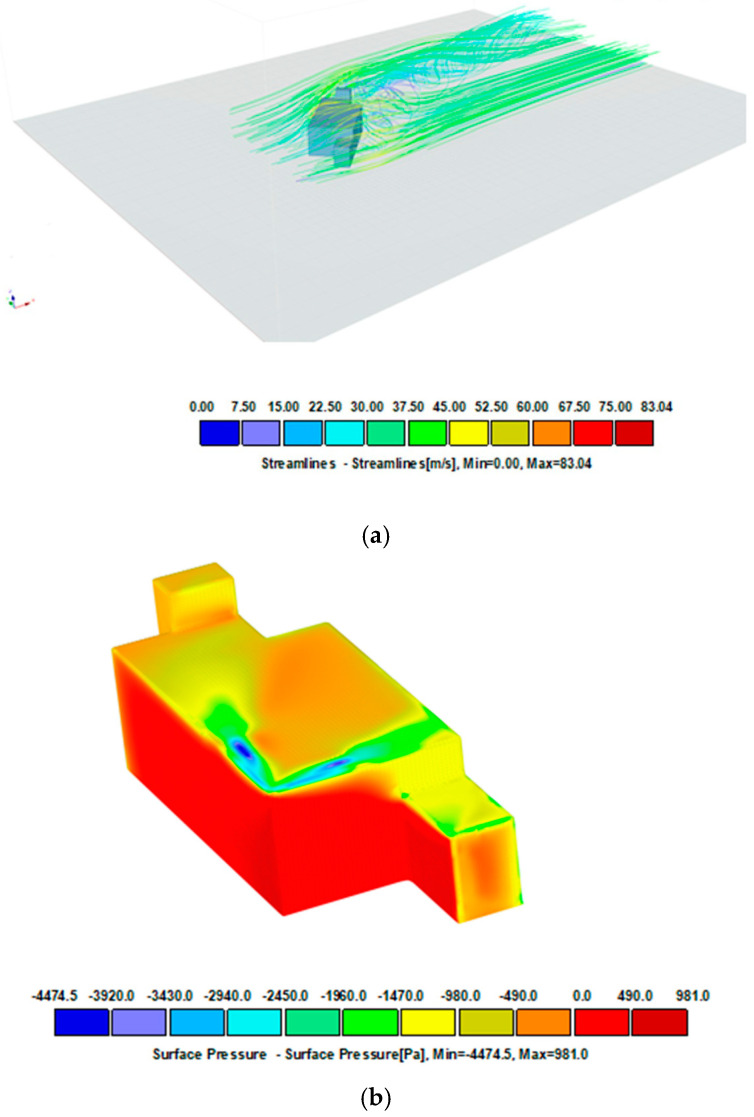
Wind velocity current lines and surface pressure. Modified model by adding attics. The load rotated by 45°. Project 2—irregular building, free stream velocity: 39.07 m/s. (**a**) Streamlines, (**b**) surface pressure.

Figure 9 shows the pressure distribution on the modified structure. Modification involves diversifying the shape of the structure, for example, by adding attics. The volume and main dimensions are similar to those in the previous example. The distribution of the pressure map in such a structure is much more diversified. The pressure value of 4.5 kPa on the roof surface is larger, but it is visible only in the edge zone. This is a considerable difference compared to the previous cases, despite the mean wind velocity and the structure’s volume and main dimensions not changing significantly. 

The technical literature [38] mentions attempts to evaluate the resistance of roof coverings to the presented operating impacts, including their dynamic nature. The values obtained in the tests are similar to those from the wind suction resistance tests described in this manuscript, i.e., several kPa.

Considering such results, one shall not forget that in actual impact conditions, the delamination of the adhesive gap formed within the roof covering typically occurs gradually, during repeated gusts of wind, to be finally pulled off during a sudden and strong gust. Therefore, it seems that the presented method for testing roof coverings’ resistance to wind impact enables an initial assessment of the parameter in laboratory conditions for the needs of specific roof covering solutions. The results can be helpful in designing building structures. The conclusion is justified by the fact that the testing method proposed by the authors for rigid structures can be applied without analysing additional dynamic impacts. 

According to the authors, the limit value taken for evaluating an adhesive bond based on the calculations according to EN 1991-1-4 [30] reflects the mean value of loads in the edge strip, up to 1.5 m wide (uniformly distributed) with no information about possible extreme values occurring only locally in this area. It should be highlighted that basic or mean wind velocities are significant but do not determine the final pressure distribution on the building surface. 

The external pressure coefficient, denoted by the symbol “Cpe”, is a dimensionless value that represents the ratio of the wind-induced pressure on the external surface of a building to the dynamic pressure of the wind.

The information summarised in the wind load standard, describing basic structures, suffices to determine the mean values. Experimental tests or CFD numerical simulations shall be used for complex geometry or non-standard load directions to obtain information about local extreme values. 

Definitely, one should not forget that the poor performance of the roof connection can be primarily attributed to the wrong selection and application of construction materials or the degradation of the strength due to ageing and long-term service within the intended useful life span [21]. 

## 5. Conclusions

This paper presents the test results of wind suction impact on roof coverings glued with polyurethane adhesives to flat roofs, that is, with slopes up to 20%. Eight sets of roof coverings were tested; concrete and trapezoidal sheets were the construction substrates. Thermal insulation layers, EPS and PIR boards, respectively, followed by flexible sheets, i.e., laminated PVC membranes and EPDM rubber-based membranes, were glued to the substrates with polyurethane adhesives. Regarding the small population of the test objects, the results cannot be generalised but should be treated as significant symptoms requiring further studies. Taking the above into account, the following conclusions can be drawn:

- The tests confirmed that some polyurethane adhesives used for the adhesive of PVC and EPDM membranes on flat roofs with an up to 20% slope ensure high resistance of complete systems to wind suction, characterised by values of up to 8 kPa (Table 3).
- The roof cover sets laid on concrete (C30/37) substrates reveal resistance to pulling off as a result of wind suction (pulling at 7.5–8.0 kPa pressure) greater than those glued to the trapezoidal sheet substrate, i.e., galvanised trapezoidal steel sheets T55 (pulling off in the pressure value range between 1.5 kPa and 7 kPa).
- A comparison of the results of wind suction resistance for laminated membranes and membranes without additional reinforcement of the surface underside reveals a significant adhesion increase in the adhesive bond when polyurethane adhesive can partly penetrate the glued layer, meaning the underside laminating layer, together forming a rigid structure. The interlayer cohesion forces within the formed adhesive bond do not exceed the adhesion values between the laminating layer and the membrane without compromising adhesion in this plane; de-bonding (pulling off) occurs in the thermal insulation layer.
- The adhesive bond formed between an EPS board and a roof covering made of a plastic (PVC) or rubber membrane (without laminating on the underside of the surface) is the weakest spot in the analysis of covering sets glued with polyurethane adhesives.
- Wind impact simulated in fatigue cycles described in this manuscript, in the 0–4 kPa range, corresponds to the averaged values occurring in a ca. 1.5 m wide range in the edge and corner zones, determined assuming extreme weather impacts on a flat roof.
- The values of the resistance of the roof coverings to wind obtained in calculations performed according to EN 1991-1-4 [30], even under stringent computational assumptions, do not reflect the actual pressure distribution, which typically has parabolic shapes and local extremes occurring at the edges of the roof.
- The external pressure coefficient plays an important role in analysing a building’s wind load; the coefficient depends on the shape, dimensions and size of the surface analysed. Basic and mean wind velocities are significant but do not determine the final pressure distribution on the building surface.
- In the case of complicated geometry and complex wind load, in order to correctly design covering solutions for a specific structure, it is recommended to compare the results obtained in the testing method described in this manuscript with the values obtained in the calculations performed to EN 1991-1-4 [30], supplemented with those determined in CFD numerical simulations.

## Figures and Tables

**Table 2:** Distribution of test cycles in a wind suction resistance test on large samples.

Number of Test Cycles	Applied Test Pressure in kPa
4	1.0
1	1.5
1	2.0
1	2.5
1	3.0
1	3.5
1	4.0

**Table 3:** Summary of tests on the roof covering resistance to wind suction.

No.	Test Variant Number	Substrate Type	Strengthening the Membrane’s Underside by Lamination	Test Result, kPa		Failure Modes
				Up to the Value of 4.0 kPa	Failure at Pressure Value	
1	I	Concrete primed with a bitumen liquid + vapour control layer (reinforced bitumen sheet) glued to the substrate over the surface	Yes	+	8.0	De-bonding of PVC membrane from PIR board
2	II			+	8.0	De-bonding of PVC membrane from EPS board
3	III			+	8.0	EPDM membrane de-bonded from PIR board
4	IV			+	7.5	EPDM membrane de-bonded from EPS board
5	V	Trapezoidal galvanised steel sheet T55		+	7.0	De-bonding of PVC membrane from PIR board
6	VI			+	7.0	De-bonding of PVC membrane from EPS board
7	VII		None	+	5.5	EPDM membrane de-bonded from PIR board
8	VIII			-	1.5	EPDM membrane de-bonded from EPS board

“+” means that tested set did not de-bond up to the value of wind suction pressure of 4.0 kPa.

## Data Availability

Data are contained within the article.
